# Research progress of remote ischemic conditioning in hemorrhagic stroke

**DOI:** 10.3389/fneur.2026.1923449

**Published:** 2026-09-09

**Authors:** Tonghu Jin, Liuyu Xu, Hao Niu, Hao Guan, Kuang Yan, Hui Shen, Aihua Liu

**Affiliations:** 1 Beijing Neurosurgical Institute, Beijing Tiantan Hospital, Capital Medical University, Beijing, China; 2 People's Hospital of Ningxia Hui Autonomous Region, Ningxia Medical University, Yinchuan, Ningxia, China; 3 Department of Cerebrovascular Disease, Neurological Disease Center, Beijing Anzhen Hospital, Capital Medical University, Beijing, China

**Keywords:** cerebral hemorrhage, hemorrhagic stroke, remote ischemic conditioning, stroke, subarachnoid hemorrhage

## Abstract

Remote ischemic conditioning (RIC) is a noninvasive, safe, and well-tolerated non-pharmacological treatment that has shown application prospects in the field of ischemic stroke. However, its application in hemorrhagic stroke is still at an early stage, the mechanism has not been fully elucidated, and clinical evidence is relatively scarce. This review found that for ICH, the mechanism of RIC may involve coordinated metabolic and immune pathways. Clinical trials have confirmed its safety, but the efficacy conclusions are inconsistent: studies involving interventions in the subacute phase have shown the potential to promote hematoma resolution, while studies involving interventions in the ultra-early phase have failed to show improvements in functional outcomes. For subarachnoid hemorrhage, the research is more preliminary, and there is no randomized controlled trial (RCT). The underlying mechanisms are thought to involve immune inflammation and vascular reactivity, while existing pilot studies have mainly focused on verifying safety rather than efficacy. In conclusion, RIC is safe when applied to hemorrhagic stroke, but its therapeutic effectiveness has not been demonstrated. Significant gaps remain, including uncertainty about the optimal timing and dosing regimens for interventions and the lack of large, multicenter trials. Future research should focus on identifying biomarkers, standardizing treatment protocols, and conducting high-quality RCTs.

## Introduction

Remote ischemic conditioning (RIC) is widely used in stroke, particularly for ischemic stroke, where its application has led to considerable breakthroughs. In recent years, a number of large-scale clinical trials have shown that RIC can improve the prognosis of patients with ischemic stroke. However, research on RIC for hemorrhagic stroke is still in its infancy, and its therapeutic mechanisms in this context are not fully elucidated. In addition, trials of treatment for hemorrhagic stroke have failed to provide a high level of evidence of treatment effect. In this review, we will present the latest information on the mechanism and clinical research of RIC treatment in hemorrhagic stroke (cerebral hemorrhage and subarachnoid hemorrhage).

Unlike ischemic stroke, hemorrhagic stroke combines abrupt tissue deformation and mass effect with a prolonged secondary injury cascade (1). In ICH, hematoma expansion, perihematomal edema, erythrocyte lysis, iron-mediated oxidative injury, blood–brain barrier disruption, and sterile inflammation contribute to neurological deterioration (2). In SAH, early brain injury during the first 72 h and subsequent delayed cerebral ischemia involve endothelial dysfunction, vasospasm and microcirculatory impairment, inflammation, and disturbed autoregulation (3). These processes provide biologically plausible targets for RIC. Brief limb ischemia can generate neural, circulating, and immune signals that may attenuate oxidative and inflammatory injury, preserve endothelial function and vascular reactivity, regulate cellular energy homeostasis and autophagy, and promote macrophage-mediated hematoma clearance. RIC is therefore being investigated not as a hemostatic treatment, but as an adjunct intended to modify secondary brain injury after, or alongside, definitive control of the bleeding source.

### Related concepts of RIC

**Figure 1:**
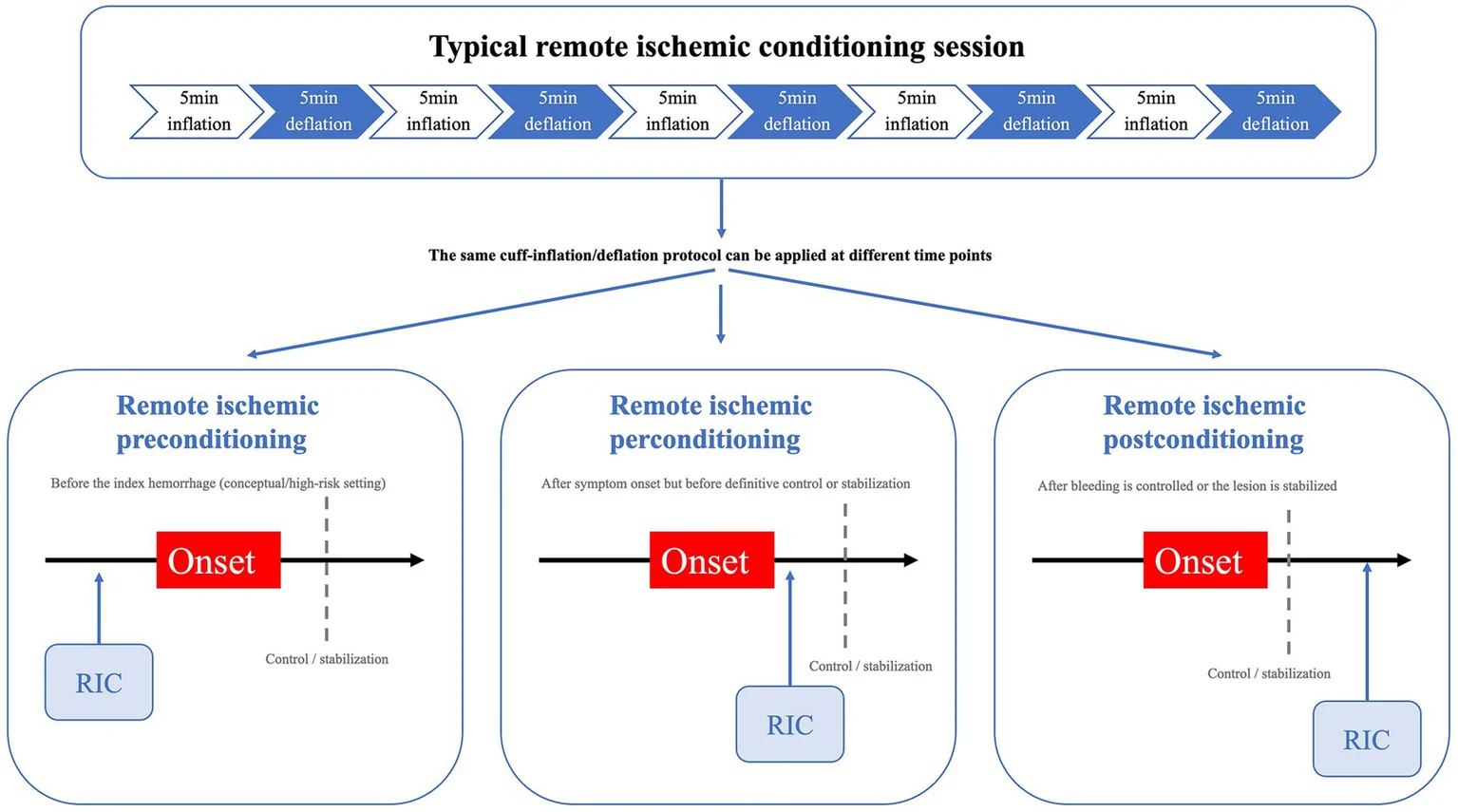
Classification and typical protocol of remote ischemic conditioning. A typical RIC session consists of four to five cycles of 5 min cuff inflation followed by 5 min deflation. In hemorrhagic stroke, the terms are defined operationally according to the timing of RIC relative to symptom onset and control or stabilization of the index bleeding event.

RIC is a simple, low-cost intervention that induces transient ischemia and reperfusion in the limb by repeatedly inflating and deflating blood pressure cuffs. This method has proven to be safe and feasible in clinical application. RIC can be categorized into three types based on the timing of the intervention: Interventions can be delivered before, during, or after the event and are called remote ischemic preconditioning, remote ischemic perconditioning, and remote ischemic postconditioning, respectively (Figure 1).

Many articles have described the mechanism of RIC, so this article will only briefly describe it. Generally, three pathways are thought to play a role in RIC protection: neurogenic, humoral, and immune-mediated pathways (4).

Ischemia is typically achieved by inflating a blood pressure cuff on one or more limbs to a pressure above the patient’s systolic blood pressure. Most intervention protocols involve four to five cycles of cuff inflation lasting 5 min. Although early clinical studies primarily used single-episode RIC administered around the time of an ischemic event, chronic remote ischemic conditioning (CRIC) is now increasingly common. This requires participants to perform daily RIC as described above for days, weeks or even months.

### Cerebral hemorrhage

According to a 2024 study, cerebral hemorrhage (ICH) accounts for 27% of all stroke events and is a common and serious type of stroke with high mortality and disability rates (5). Long-term functional decline, cognitive decline, and post-ICH dementia are common sequelae after an ICH event, leading to a decreased quality of life (6–8).

### Pathophysiological targets and therapeutic challenges in ICH

Hematoma enlargement is consistently associated with poor functional outcome in ICH patients (9); Perihematoma edema (PHE) secondary to ICH, which peaks at days 7–10 post-ICH, is thought to be independently associated with poor functional outcome (10); Therefore, hematoma evacuation and PHE treatment are considered to be the key to the treatment of ICH, but there is no effective treatment to significantly improve the prognosis of patients. In recent years, several studies have been performed, but high-quality randomized clinical trials of craniotomy for hematoma evacuation and minimally invasive surgical thrombolysis have not yet shown positive results in terms of functional recovery (11).

### RIC’S basic mechanism research on ICH

**Figure 2:**
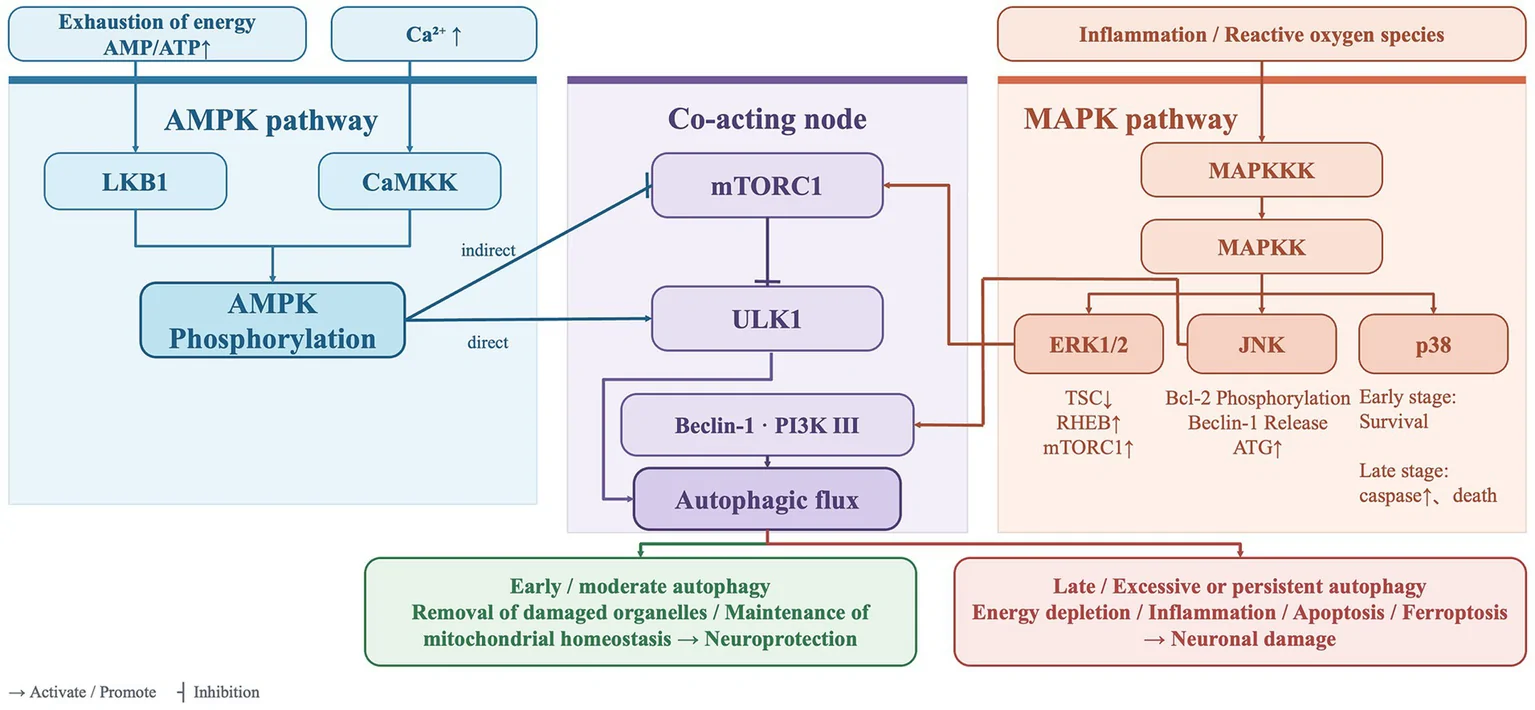
The relationship between AMPK and MAPK pathways.

Recent studies suggest that RIC exerts multifaceted neurovascular protection after ICH through coordinated activation of metabolic and immune pathways rather than a single cascade. The MAPK and AMPK pathways, which have been identified in experimental ICH models, represent two major nodes of this regulatory network. MAPK activation promotes the expression of cytoprotective proteins such as HO-1 and transferrin, enhancing antioxidant defense and iron metabolism (12), while AMPK activation modulates cellular energy homeostasis and promotes macrophage-mediated hematoma clearance (13). Notably, these two pathways are not independent: they have both collaborative cooperation and complex cross-disciplinary dialogues of mutual restraint, jointly and precisely regulating the autophagy, survival and death fate of cells (14). The schematic diagram (Figure 2) illustrates the interplay between AMPK and MAPK pathways in mediating RIC-induced protection. Importantly, excessive or sustained MAPK activation can lead to neuronal apoptosis; AMPK acts as a negative regulator in this context, preventing overactivation of the MAPK cascade and maintaining the balance between autophagy and apoptosis.

These mechanistic claims should nevertheless be interpreted cautiously. Most evidence comes from animal models, and some pathways are inferred from ischemic rather than hemorrhagic stroke. The direction and magnitude of MAPK, AMPK, and autophagy responses depend on cell type, injury phase, and stimulus intensity. A mechanistic effect on hematoma clearance or edema therefore cannot be assumed to translate into better functional outcome.

### RIC’S clinical research on ICH

Zhao et al. (15) published the clinical trial of “RICH-1.” RIC achieved positive results in the study of 40 patients (who received RIC every day for 7 days after ICH): None of the patients experienced RIC-related neurological deterioration or adverse events within 7 days. 7 days later, compared with the baseline, there was no statistically significant change in hematoma volume between the RIC group and the control group, but compared with the control group, the hematoma regression rate in the RIC group was significantly higher. The relative PHE in the RIC group was significantly lower than that in the control group. It can be concluded that RIC therapy is safe and tolerable for patients with ICH. It may increase the rate of hematoma dissolution and reduce hematoma (15). However, the trial generated a biological signal, but its small sample, lack of sham control, and reliance on imaging surrogates limit causal and clinical interpretation.

Blauenfeldt et al. (16) published the research results of RESIST, which included 165 patients with cerebral hemorrhage and found no correlation between RIC intervention and improvement in functional outcomes. Such results may be related to multiple factors. First, all types of stroke patients were included in this study; Secondly, this study only performed RIC intervention on one upper limb. Thirdly, the degree of stroke in the included patients was relatively mild.

Kakarla et al. (17) published the clinical trial results of “ROCOCHET.” Due to limitations such as a small sample size, high withdrawal rate, and an insufficient number of RIC interventions, this study did not confirm that RIC could reduce PHE in ICH patients. However, it proposed that early RIC treatment for ICH patients is safe and feasible.

In 2026, RICH-2 provides the strongest disease-specific evidence. This phase 3, multicenter, randomized, sham-controlled, outcome-blinded trial enrolled 458 patients at 20 centers with supratentorial ICH of 10–30 mL, moderate neurological deficits, no surgical indication, and RIC initiated 24–48 h after onset. Favorable 90-day outcome (mRS 0–2) occurred in 68.1% of the RIC group and 71.2% of the sham group, with no significant benefit (18). RICH-2 did not demonstrate functional efficacy of RIC in the overall population of non-surgical patients with 10–30 mL supratentorial ICH, but it confirmed an effect on haematoma clearance and identified a prespecified signal of possible functional benefit in patients with a baseline haematoma volume greater than 20 mL.

Although these RIC studies on cerebral hemorrhage verified the safety of RIC intervention, no positive results of therapeutic effect were obtained. This might be caused by multiple factors: First, insufficient induction of RIC: The duration of RIC is too short, and RIC is only performed on one upper limb; Secondly, the prognosis of the included patients was relatively good, and the improvement of RIC intervention was not obvious. Thirdly, the sample size is insufficient to meet the statistical requirements.

The differences in the results of these studies may be closely related to the choice of intervention time (Table 1). In the RICH-1 study, RIC was initiated 24–48 h after ICH (subacute phase) and showed efficacy in promoting hematoma absorption and resolution. This time window coincides with the peak of the endogenous hematoma clearance mechanism, which is thought to enhance this clearance process through AMPK-dependent immunomodulation. In sharp contrast, both the RICOCHET and RESIST studies implemented RIC in ultra-early time Windows (within 6 h after symptom onset and in the prehospital period, respectively). Although early intervention has some justification from the perspective of neuroprotection, RIC applied only at this early stage may miss the key window of action in the later stage - that is, the stage when RIC promotes hematoma regression and thus has a significant effect on the outcome of ICH. Moreover, in ischemic stroke, especially in the context of the RESIST study combined with reperfusion therapy, RIC alone may not be sufficient to counter the intense injury cascade in the acute phase. In addition to the time factor, the study population and the design of the primary end point were critical (Table 1). The RICH-1 study focused on a homogeneous ICH population with moderate hematoma volume and used sensitive imaging biomarkers directly related to the mechanism of intervention as a means of evaluation. In contrast, the RESIST study included a broad population that included both ischemic and hemorrhagic strokes, which may have diluted specific treatment signals for a given pathology; At the same time, the assessment of functional end points may be limited by a “ceiling effect” in the population of patients with minor strokes, which may affect the results. Meanwhile, the RICH-2 study suggests that, despite the negative overall functional outcome, patients with larger hematomas may represent a potentially advantageous population that warrants further validation.

**Table 1:** Comparison of RIC in ICH studies.

Study	RICH-1	RICOCHET	RESIST	RICH-2
Population	ICH	ICH	Stroke (including ICH)	ICH
Study design	proof-of-concept, assessor-blinded, pilot open-label randomized controlled trial	prospective randomized, controlled, single-center, interventional blinded endpoint study	multicenter, randomized, patient and outcome-assessor blinded, sham-controlled clinical trial	randomised, multicenter, sham-controlled, outcome-blinded trial
Sample size	40	60	1,500	458
Time	24 to 48 h after onset	Within 6 h of onset	Prehospital	24 to 48 h after onset
Air bag pressure	200 mmHg	200 mmHg	200 mmHg(Or 35 mmHg above SBP)	200 mmHg
A single cycle	5 min ischemia / 5 min reperfusion	5 min ischemia / 5 min reperfusion	5 min ischemia / 5 min reperfusion	5 min ischemia / 5 min reperfusion
Number of cycles per treatment	5	4	5	5
Frequency of treatment	Once daily	Twice daily	Twice daily	Once daily
Total course of treatment	7 days	7 days	7 days	7 days
Selection of limbs	Unilateral upper limb	Bilateral upper limbs	Unilateral upper limb	Unilateral upper limb
Primary outcomes	Hematoma absorption rate and edema	Edema extension distance	Functional outcome at 90 days (mRS)	mRS 0–2 at 90 days
	Positive	Negative	Negative	Negative

### Subarachnoid hemorrhage

Subarachnoid hemorrhage (SAH) is the third most common type of stroke and is usually associated with aneurysm rupture (19). Despite the decreasing trend in incidence, SAH still affects about 8 per 100,000 people annually (20). Up to one-third of SAH survivors experience reduced quality of life, attention deficits, cognitive impairment, depression, and more (21, 22).

### Pathophysiological targets and therapeutic challenges in SAH

The mechanisms leading to poor outcomes in SAH are complex and multifactorial, involving early brain injury (EBI) and a range of different subsequent processes, such as delayed cerebral ischemia (DCI). EBI, a complex pathological process occurring within 72 h after SAH, is a primary driver of poor prognosis and has received increasing attention in recent years. Effective preventive strategies and treatments for cerebral vasospasm and DCI are lacking. Survivors often struggle with residual neurologic deficits that seriously affect their quality of life. Furthermore, these patients face a 15-fold higher risk of subsequent bleeding events than the general population and experience increased long-term mortality, mainly due to cardiovascular and cerebrovascular diseases (23, 24).

A number of previous clinical studies have been conducted (25–31), but the vast majority have ended in negative results, and to this day, nimodipine remains the only drug approved by the FDA for SAH.

### Mechanistic rationale for RIC in aSAH

Research on RIC for SAH is still in an early stage. Mechanistic studies have highlighted the therapeutic potential of RIC in SAH management.

Compared with ICH, RIC in SAH appears to act primarily through modulation of vascular reactivity and immune–inflammatory homeostasis. Epigenetic profiling studies revealed that RIC can alter DNA methylation and gene expression of key regulators involved in the cell cycle and inflammatory responses, implying a systemic reprogramming effect (32); However, this study enrolled only 13 patients, had limited statistical power, and did not rule out potential effects of other treatment modalities on gene expression. At the cellular level, RIC has been shown to reduce cerebral vasospasm and preserve endothelial function (33), partly through STAT3/STAT5 signaling balance and the regulation of Th17/Treg cell differentiation. In this context, RIC may act upstream of multiple signaling pathways: STAT5 activation enhances Foxp3-positive Treg expansion, whereas inhibition of STAT3 suppresses Th17-driven inflammation, jointly attenuating EBI and subsequent DCI (34). These pathways provide a plausible immune-vascular framework in which RIC can act upstream of early brain injury and delayed cerebral ischemia. However, most pathway data are preclinical, and no clinical trials have shown that changes in immune or endothelial biomarkers mediate improved neurologic outcomes.

Taken together, RIC’s protective mechanism in SAH is characterized by immune–vascular crosstalk: systemic immune rebalancing via STAT3/STAT5 signaling translates into improved cerebral vascular stability. Targeting this axis may open new therapeutic directions for preventing DCI and cognitive decline after SAH.

### RIC’S clinical research on SAH

The clinical research of RIC in the treatment of SAH is still in its infancy, and no randomized controlled trial has been conducted (Table 2). Koch et al. (35) performed RIC treatment on 33 patients with SAH and found that RIC treatment was safe and feasible for SAH patients, and was well tolerated by patients. Gonzalez et al. (36) performed 75 successful RIC treatments in 20 patients in 2014, and no patient developed deep vein thrombosis. These two early studies mainly focused on the safety of RIC treatment for SAH patients, and explored the possibility of RIC treatment for SAH patients. Kim et al. (37) enrolled 1,043 SAH patients from multiple centers, and proposed that there might be an endogenous protective mechanism against cerebral vasospasm by examining the presence of previous stenotic occlusive cerebrovascular disease (CVD) and/or previous cerebral infarction and establishing a model. Laiwalla et al. (38) matched 21 aSAH patients treated with RIC with 61 control patients and showed that RIC intervention may improve the neurocognitive function of patients and reduce the incidence of stroke and mortality. Xu et al. (39) performed RIC intervention on 30 aSAH patients, and detected their coagulation function, deep vein thrombosis, and cerebral blood flow. Compared with the control group, there was no significant difference in coagulation function and cerebral blood flow of patients, and no deep vein thrombosis of compressed limbs was detected, which verified the safety of RIC intervention in aSAH patients. Raval et al. (40) published a prospective intervention study of RIC application in patients with aSAH, in which two groups of patients (11 in each group) were treated with High-Pressure Occlusion and Low-Pressure Occlusion, respectively. It was also matched with 11 retrospective control patients to verify its safety and point out that RIC intervention may shorten the length of hospital stay in patients with aSAH. These studies explored the tolerability and safety of RIC in the treatment of SAH patients without further validation of its efficacy.

**Table 2:** Comparison of RIC in SAH studies.

Study (year)	Time	Pressure	A single cycle	Number of cycles per treatment	Frequency of treatment	Total course of treatment	Limb selection	Primary outcomes
Koch et al. (35) (2011)	Within 96 h of ictus	200 mmHg (or >SBP 20 mmHg)	5 min ischemia/5 min reperfusion	3	Every 24–48 h	14 days or discharge	Lower limb	Safety & feasibility: well-tolerated, no neurovascular injury.
Gonzalez et al. (36) (2014)	Within 72 h (majority)	>SBP 20 mmHg (Doppler confirmed)	5 min ischemia/5 min reperfusion	4	Every other day	4 sessions	Lower limb	Safety & feasibility: no limb injury/DVT; no DIND during enrollment.
Laiwalla et al. (38) (2016)	Within 2–12 days	>SBP 20 mmHg (Doppler confirmed)	5 min ischemia/5 min reperfusion	4	Every other day	4 sessions	Lower limb	Efficacy: associated with good outcome (mRS 0–2), OR = 5.17.
Xu et al. (39) (2020)	Within 72 h	180 mmHg (or >SBP 20 mmHg)	5 min ischemia/5 min reperfusion	5	Daily (over 7 days)	5 sessions	Upper or lower limb	Safety: no significant effect on coagulation/CBF; no DVT or new infarction.
Raval et al. (40) (2021)	Within 72 h of onset	>SBP 20 mmHg	5 min ischemia/5 min reperfusion	4	Every other day	Up to 14 days or ICU discharge	Lower limb	Efficacy: shorter hospital/LOS in LPO vs. Control. No difference in vasospasm/infarction.

At present, the evidence of aSAH mostly comes from small phase I studies, pilot trials and observational cohorts. Safety and feasibility are supported by reasonable evidence, but efficacy is uncertain and unproven. A positive signal observed early on May be limited by small sample sizes, selective reporting, multiple comparisons, and noncontemporated controls. The results of randomized controlled trials and meta-analyses are neutral and do not support the use of RIC for patients with aSAH outside of routine clinical practice.

### Future perspectives and research priorities

Future research should shift from extensive proof-of-concept to mechanism-oriented disease-specific trial designs.

Identify the timing of intervention related to bleeding control. For ICH, trials should report time to symptom onset, baseline and repeat imaging findings, hematoma stability, and time from symptom onset to RIC. For aSAH, aneurysm fixation should usually be completed before RIC is repeated, and protocols should specify whether treatment is directed at early brain injury, delayed cerebral ischemia window, or both. The hyperacute and subacute strategies should not be analyzed together as biologically equivalent.

Establishing an effective biological dose. Formal dose-finding studies should compare limb selection, individualized cuff pressure, number of cycles, once-daily versus twices-daily treatment, and total treatment courses. Device documentation of adherence and confirmation of complete limb occlusion were required. Without adequate target engagement, negative trials will fail to distinguish mechanism ineffectiveness from inadequate stimulation.

Enrich the population and standardize the outcome measures. ICH studies should predetermine hematoma volume, location, perihematoma edema burden, indication for surgery, and baseline severity. aSAH studies should be stratified according to Hunt-Hess or WFNS grade, modified Fisher grade, treatment modality, and baseline risk of delayed ischemia. Core outcomes should include blinded 90-day modified Rankin scale scores, mortality, and a standardized hematoma /PHE measure for ICH, aSAH consensus definition of delayed cerebral ischemia, and MRI detected infarction.

Demonstrate target involvement. A range of blood and imaging biomarkers should be included, with candidates including monocyte/macrophage phenotypes, inflammatory cytokines, HO-1-associated proteins, endothelial function, circulating micrornas or exosomes, hematoma clearance kinetics, perfusion imaging, and diffusion-weighted MRI. Mediation analyses should validate biological pathways of change between RIC exposure and clinical outcomes.

A sham control platform with sufficient power was used. Future efficacy trials need to use concealed trial-group assignments, credible sham procedures, blinded outcome assessments, prespecified subgroup analyses, complete adverse-event reporting, and statistical power for disease-specific end points. Adaptive or factorial design can effectively compare time and dose, and avoid repeating small studies with insufficient power.

## Conclusion

While the progress of RIC in ischemic stroke has been remarkable, its application in hemorrhagic stroke still has a long way to go. Although RIC is generally considered safe, there are concerns that it may aggravate the condition in hemorrhagic stroke patients—especially in the acute phase—by affecting blood pressure or hemodynamics. Therefore, previous RIC studies in this field have often focused on the safety of interventions. First, in terms of basic mechanism research, although some studies have proposed that RIC may play a role through a variety of mechanisms such as immune regulation, angiogenesis, and neuroplasticity, these mechanisms are not completely clear and need to be further studied. Promising candidates include circulating microRNAs, exosome-derived proteins involved in oxidative stress and inflammation (such as HO-1 and IL-10), and metabolomic indicators reflecting mitochondrial and endothelial function. In addition, to better replicate the clinical heterogeneity of hemorrhagic stroke, preclinical studies should employ animal models incorporating common comorbidities such as hypertension, diabetes, and aging, which are known to alter vascular reactivity and immune responses. Such models would provide more translational insights into RIC’s safety, optimal timing, and treatment duration in real-world patients. Secondly, in terms of clinical research, most of the studies are exploratory studies with small sample sizes, and there is no multicenter randomized controlled trial with large sample sizes to verify RIC for improving neurological function in hemorrhagic stroke. Finally, RIC intervention protocols vary greatly among studies: the number, duration, pressure and other parameters of ischemia and reperfusion are different. There is still no clear and unified standard for intervention mode, dose and timing, and the best treatment plan is still unknown. For patients with hemorrhagic stroke, much work remains to be done before RIC can be widely accepted.
